# An update on the ocular phenotype in patients with pseudoxanthoma elasticum

**DOI:** 10.3389/fgene.2013.00014

**Published:** 2013-04-04

**Authors:** Martin Gliem, Julie De Zaeytijd, Robert P. Finger, Frank G. Holz, Bart P. Leroy, Peter Charbel Issa

**Affiliations:** ^1^Department of Ophthalmology, University of BonnBonn, Germany; ^2^Department of Ophthalmology, Ghent University Hospital, Ghent UniversityGhent, Belgium; ^3^Centre for Medical Genetics, Ghent University Hospital, Ghent UniversityGhent, Belgium

**Keywords:** pseudoxanthoma elasticum, angioid streaks, retina, Bruch’s membrane, choroidal neovascularization

## Abstract

Pseudoxanthoma elasticum (PXE) is an inherited multi-system disorder characterized by ectopic mineralization and fragmentation of elastic fibers in the skin, the elastic laminae of blood vessels and Bruch’s membrane in the eye. Biallelic mutations in the ATP-binding cassette (ABC) transporter gene *ABCC6* on chromosome 16 are responsible for the disease. The pathophysiology is incompletely understood. However, there is consent that a metabolic alteration leads to dysfunction in extracellular calcium homeostasis and subsequent calcification of connective tissues rich in elastic fibers. This review summarizes and aims at explaining the variety of phenotypic ocular findings in patients with PXE. Specialized imaging techniques including white light fundus photography, blue light autofluorescence, near-infrared confocal reflectance imaging, high resolution optical coherence tomography, fluorescein and indocyanine green (ICG) angiography have revealed characteristic lesions at the ocular fundus of PXE patients. These include the classic signs of angioid streaks, peau d’orange, comet lesions, and choroidal neovascularizations (CNVs), but also the more recently recognized features such as chorioretinal atrophy, subretinal fluid independent from CNV, pattern dystrophy-like changes, debris accumulation under the retinal pigment epithelium, reticular drusen and a decreased fluorescence on late phase ICG angiography.

## HISTORICAL NOTES AND TERMINOLOGY

Angioid streaks, one of the most striking ocular findings in patients with PXE, were first reported in a case presentation by Robert Doyne from the Oxford Eye Hospital in 1889 as *irregular jagged lines* (Doyne, 1889). The presented patient had a history of blunt trauma to both eyes, and the streaks were interpreted as “*rupture to the pigment layer of the retina*.” In 1891, the German ophthalmologist Otto Plange from Münster independently reported a patient he observed during his residency in Bochum (Plange, 1891). In his extensive description of angioid streaks, he interpreted them as intraretinal pigmented deposits following hemorrhage. Due to their “*appearance of an obliterated system of blood-vessels*,” Knapp subsequently coined the term *angioid streaks* (Knapp, 1892). In their descriptions, Plange and Knapp also mentioned that “*streaks seemed to fade within a mottled area*” (Plange, 1891), possibly representing what we now call peau d’orange. It took more than two further decades before Kofler, based on meticulous clinical observations, eventually suggested that angioid streaks are due to breaks in BM (Kofler, 1917). His observations were later supported by histological studies (Böck, 1938; Hagedoorn, 1939; Verhoeff, 1948; McWilliam, 1951; Jensen, 1977; Dreyer and Green, 1978) and were more recently confirmed by *in vivo* imaging (Charbel Issa et al., 2009). In 1941, Scholz provided an excellent thorough review of the early literature on angioid streaks, analyzing a total of 188 reported cases (Scholz, 1941).

The term *peau d’orange* was suggested by Smith and colleagues in 1964 to describe the mottled fundus appearance (Smith et al., 1964). Gass (2003) suggested the term “comet tail lesions” for the characteristic spot-like chorioretinal lesions with a tail pointing toward the posterior pole.

As pointed out by Hagedoorn (Hagedoorn, 1939), Hallopeau and Laffitte were the first to report a possible relation between PXE and retinal disease (Hallopeau and Laffitte, 1903). However, it took until 1929 for the Swedish ophthalmologist Ester Grönblad together with the dermatologist James Strandberg to recognize the syndromic association between the characteristic ocular and skin phenotypes (Grönblad, 1929; Strandberg, 1929). Although the term PXE was originally introduced in 1896 to specifically describe the dermal pathology, it is generally accepted to use the term synonymously with Grönblad–Strandberg syndrome.

## FUNDUSCOPIC FINDINGS

**FIGURE 1:**
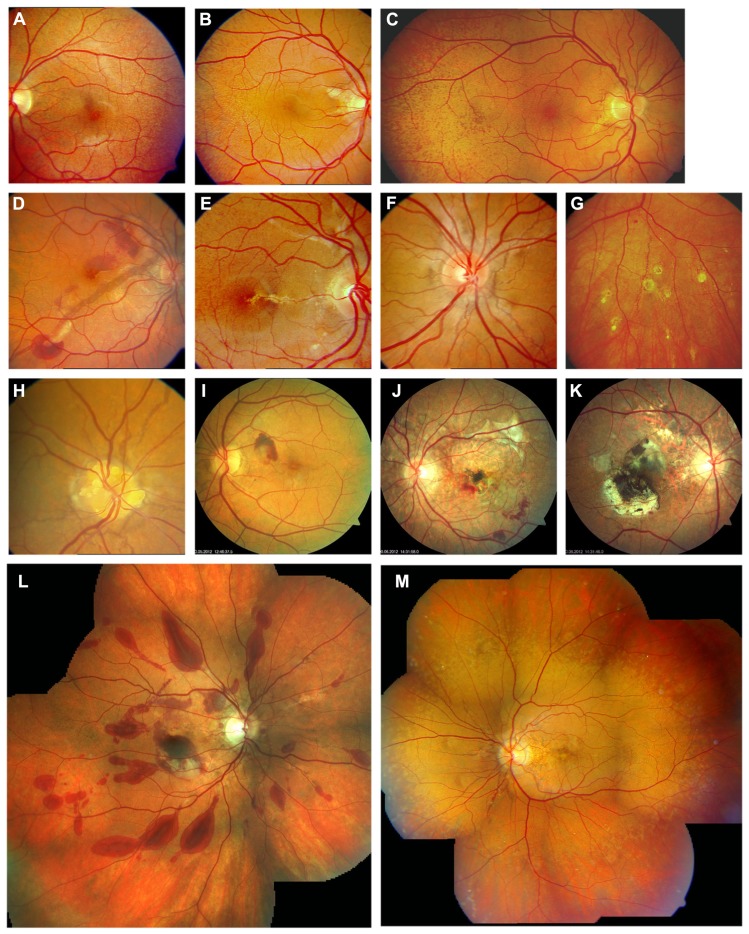
**Clinical features of pseudoxanthoma elasticum on funduscopic examination**. Peau d’orange is characterized by small dark spots on a whitish or opaque background **(A–C)** and likely represents the transition between calcified and non-calcified Bruch’s membrane. Peau d’orange appears to begin at the posterior pole **(A)**, spreading peripherally over time **(B,C,M)**. Peau d’orange is most pronounced temporaly but may be visible circular within the retinal periphery **(M)**. Angioid streaks are reddish or brownish irregular lines that often form a peripapillary ring from where they radiate into the periphery **(D–F,H,M)**. Small roundish chorioretinal atrophies are frequently found in the retinal periphery eccentric to peau d’orange, but may also occur closer to the optic nerve head. They often present with a tail pointing toward the optic nerve head, leading to the descriptive term of comet tail lesions **(G)**. Not a pathognomonic but frequent finding are optic disc drusen **(H)**. Angioid streaks may be complicated by the development of choroidal neovascularizations **(D,I)** leading to subsequent atrophy and scarring **(J,K)**. Progressive chorioretinal atrophy may also enlarge without presence of active neovascularizations **(J)**. The resistance of Bruch’s membrane to ocular trauma is reduced leading to extensive bleeding after minor traumata **(L)**. PXE-associated fundus features are summarized in **(M)** with peau d’orange encompassing the circular periphery, comet tail lesions within the periphery, Angioid streaks not exceeding peau d’orange and central pattern dystrophy-like changes.

Pseudoxanthoma elasticum retinopathy (**Figure 1**) is characterized by a mottled aspect of the temporal retinal midperiphery, called peau d’orange, and angioid streaks, representing ruptures in BM. The latter may lead to subretinal choroidal neovascularization (CNV) with a risk for spontaneous hemorrhage and scar formation with subsequent loss of vision. In addition, comets and comet tail lesions in the midperiphery with a variable degree of RPE atrophy represent a unique sign of the condition (Gass, 2003). The clinical heterogeneity, inherent to PXE, is also reflected in the retinal phenotype: although invariably present, the retinopathy remains rather limited in some patients, making an early diagnosis more difficult, whereas in others the phenotype is severe.

Retinal involvement generally increases with age. Due to the high phenotypic variability (Plomp et al., 2009) even within families and the large number of mutations and polymorphisms in the *ABCC6* gene, no clear genotype–phenotype correlations have been established. As well, no clear correlations between severity of the ocular phenotype and that of other PXE-related manifestations, such as cardiovascular disease, have so far been identified. A minor retinal phenotype, limited to comet lesions, was observed in 8 of 25 carriers of ABCC6 mutations in one study (De Zaeytijd et al., 2010).

Calcification in BM predisposes to breaks within this membrane most likely through the physiological tensions exerted on the eye by the extraocular muscles, with a point of convergence at the optic nerve head. Moreover, even minor trauma to the eye that otherwise would not have a major effect on ocular morphology and function, may cause retinal hemorrhage independent from presence of a CNV (**Figure 1L**) and/or development and growth of angioid streaks (Britten, 1966; Hagedoorn, 1975). Therefore, activities with potential trauma to the eye should be avoided, or adequate protection should be worn. Calcification of BM may also impede the exchange of nutrients, growth factors, and waste products between RPE and choroid, eventually leading to functional and structural compromise of the RPE, the choriocapillaris and the retina.

### PEAU D’ORANGE

**FIGURE 2:**
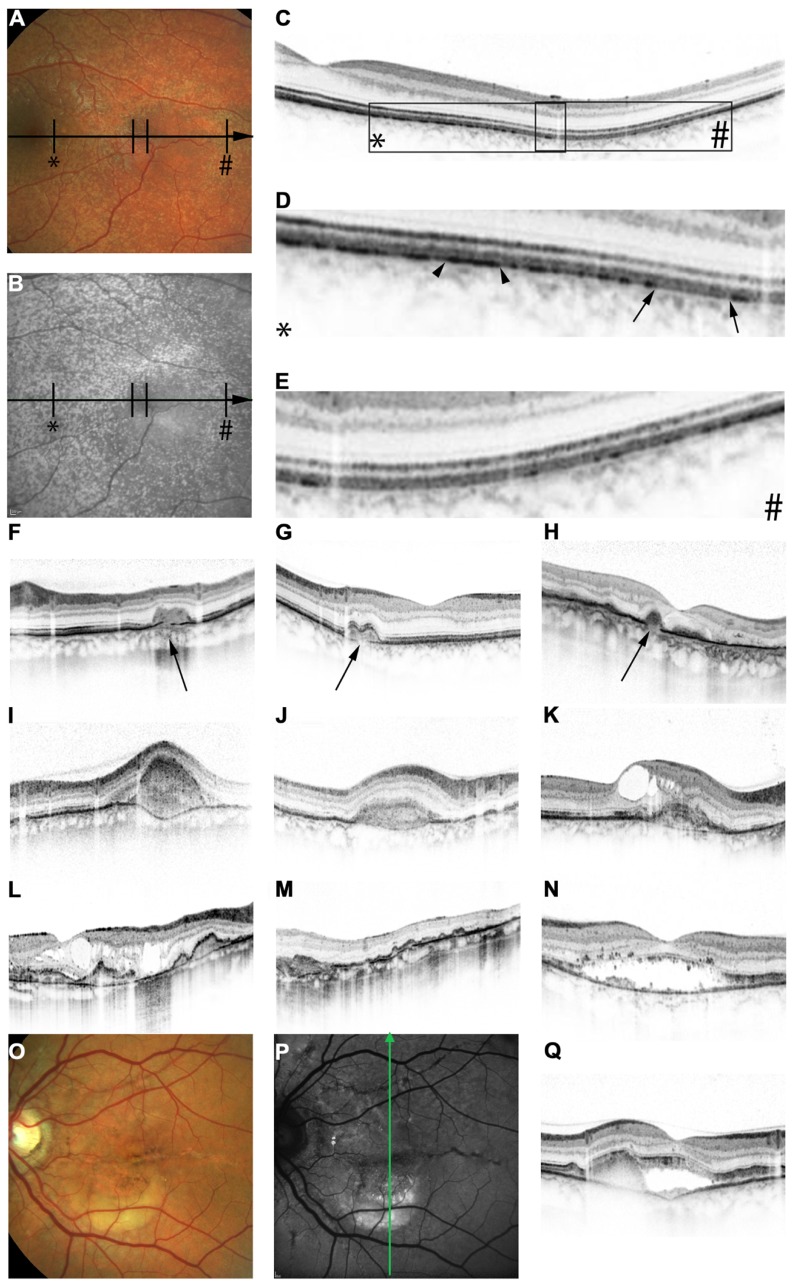
**Features of pseudoxanthoma elasticum on optical coherence tomography**. Calcification of Bruch’s membrane may be seen on OCT images. This is best illustrated within areas of peau d’orange, the transition zone between calcified and un-calcified Bruch membrane **(A–E)**. The horizontal arrow in **(A)** and **(B)** indicates the placement of the OCT scan in **(C)**. 2.5x magnifications of characteristic details in **(C)** are shown in **(D,E)**. Area * corresponds to **(D)** and area # to **(E)**. Areas of increased reflectivity within the outer zone of RPE-Bruch’s membrane complex (arrow heads in **D**) correlate to the whitish opaque fundus reflex on color images **(A)** and the increased signal on near-infrared reflectance images **(B)**. Areas of lower reflectivity (**E**, arrows in **D**) correlate to the normal fundus reflex. Angioid streaks correlate to breaks within the thickened and hyperreflective Bruch’s membrane (**F–H**, arrows). Fibrovascular tissue may grow through such breaks **(I,J)**. A typical complication of angioid streaks is the development of choroidal neovascularizations leading to retinal exsudation **(K)**. Eventually, atrophy of the retinal pigment epithelium is associated with atrophic changes in the photoreceptor layer with **(L)** or without **(M)** cystoid retinal lesions. In some patients there may be persistent subretinal fluid independent of choroidal neovascularizations **(N,Q)**. If longstanding, a vitelliform lesion may present with deposition of yellowish hyperautofluorescent material at the bottom of the lesion **(O–Q)**. The green arrow in **(P)** indicates the placement of the OCT scan in **(Q)**.

Peau d’orange appears to be the earliest funduscopically visible alteration in patients with PXE, preceding the development of angioid streaks (Gills and Paton, 1965; Krill et al., 1973; Naouri et al., 2009). Peau d’orange is characterized by small dark spots, within an area of a slightly whitish or opaque fundus reflex (**Figures 1A**–**C,M, 2A**, and **6K**). This pattern may be observed at the posterior pole very early in the disease and more peripheral in later disease stages. In the latter case, the slightly whitish or opaque fundus reflex may have become more uniform posteriorly to the area of peau d’orange. Peripheral to the peau d’orange region, the fundus reflex is usually darker (**Figure 1M**). The difference between the central and peripheral fundus reflex is more obvious in dark pigmented patients in whom the higher overall fundus pigmentation contrasts better with the more posteriorly located whitish areas. It may remain unnoticed in less pigmented individuals as well as in very late disease stages (Charbel Issa et al., 2010a).

Bruch’s membrane is believed to be the primarily affected anatomic structure of the ocular fundus in patients with PXE. Anatomical differences in thickness and integrity between central and peripheral BM (Chong et al., 2005) may account for a higher vulnerability and thus earlier calcification at the posterior pole, with a subsequent centrifugal disease spread.

Based on clinical observations, it has been hypothesized that peau d’orange represents a visible transition zone of BM calcification (Charbel Issa et al., 2010a). Replacement of peau d’orange by a more uniform area of whitish fundus reflex posteriorly may occur once calcification has become continuous. A centrifugal spread of calcification, with the leading edge representing the transition zone of calcifying BM, appears to be the rational for the observation that peau d’orange is seen increasingly more peripheral with age. Currently, there is little published longitudinal data to confirm such cross sectional clinical observations.

Peau d’orange often is assumed to be most pronounced and widespread in the temporal midperiphery. However, there is good evidence that this characteristic fundus finding simply is the most prominent manifestation of a transition zone that in actual fact is present circumferentially, though not symmetrically (Charbel Issa et al., 2010a; **Figures 1M** and **5**).

### ANGIOID STREAKS

Angioid streaks, which are breaks in BM, are the most obvious and most consistently observed funduscopic finding in patients with in PXE (**Figures 1D**–**F,H,M**, and also visible in most other figures).

Angioid streaks present ophthalmoscopically as irregular and jagged lines that radiate from a concentric peripapillary ring toward the equator of the eye. Visibility and colour of angioid streaks may depend upon the degree of secondary alterations, such as loss of the choriocapillaris, depigmentation or loss of RPE cells, or fibrosis. In some patients, they may be limited to a few almost imperceptible lines whereas in others they present as a complicated interlacing network or grid. The streaks are most prominent at the posterior pole of the eye and typically taper and fade toward the equator of the eye, often dividing into smaller branches. Occasionally they continue for a short distance as irregular, white, depigmented lines. Pigmented wing-like hyperpigmentations may be found along angioid streaks.

Alterations in the RPE adjacent to streaks commonly occur. Loss of pigment imparts a “feathered” appearance to the streak (De Zaeytijd et al., 2010). Angioid streaks enlarge in length and width over time (Mansour et al., 1993), and usually do not cross areas of peau d’orange (Charbel Issa et al., 2010a). The latter finding supports the notion that calcification of BM predisposing to angioid streak formation is present centrally from peau d’orange. In addition, stress lines on the posterior pole of the eye converge on the optic nerve head, which is both the anchor point for the optic nerve, and the hinge point at which, despite the flexibility of the optic nerve itself, a certain degree of mechanical tilting of the eyeball occurs, relative to the optic nerve, in a direction dependent on the gaze. The combination of these stress lines in a calcified BM probably leads to angioid streak formation.

Angioid streaks are usually obvious on funduscopy. Confocal near-infrared (NIR) reflectance imaging was found to be superior to other imaging modalities (Charbel Issa et al., 2009; De Zaeytijd et al., 2010) to document angioid streaks (Charbel Issa et al., 2009). In areas of widespread chorioretinal atrophy, angioid streaks are usually not visible anymore. However, breaks in BM may still be detected in such cases using spectral domain optical coherence tomography (SD-OCT; Charbel Issa et al., 2009).

Although other ocular diseases such as high myopia and ocular trauma may also lead to breaks in BM, the pattern of break formation is different in those diseases (Pruett et al., 1987). While myopic lacquer cracks are short and usually found in a reticular distribution within a posterior staphyloma, traumatic tears are characteristically curved parallel to the optic disk margin and are usually located temporal to the disk in only one eye.

Angioid streaks are not necessarily associated with a noticeable decrease in retinal function, and visual acuity may remain normal even in presence of a streak crossing the fovea. Angioid streaks may occur with or without overt damage to the RPE (Charbel Issa et al., 2009). Probably, the overlying RPE first needs to be compromised before loss of function occurs. However, high resolution structure–function correlations, e.g., with microperimetry, has only recently become available (Charbel Issa et al., 2010b) and has not yet been performed in patients with PXE. Definite vision loss occurs when angioid streaks are complicated by the development of a CNV or atrophy of the RPE.

### PERIPHERAL COMET AND COMET TAIL LESIONS

**FIGURE 3:**
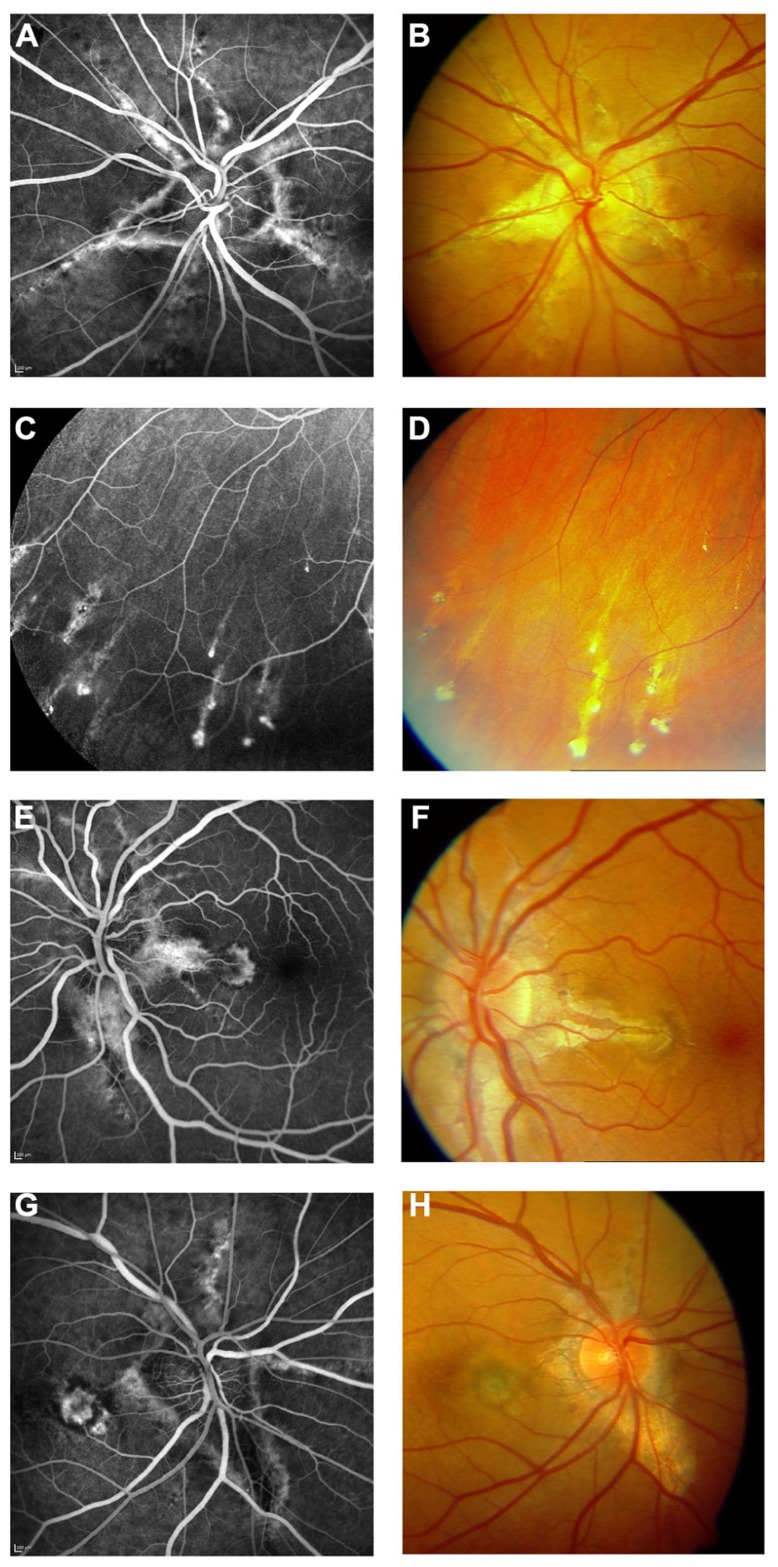
**Features of pseudoxanthoma elasticum on fluorescein angiography**. Angioid streaks typically show a variable staining on fluorescein angiography **(A,B)**. Comet tail lesions appear as hyperfluorescent spots with their tail toward the optic disk **(C,D)**. Choroidal neovascularizations are mostly classic membranes. Sometimes, their detection may be difficult due to adjacent staining of angioid streaks **(E–H)**.

**FIGURE 4:**
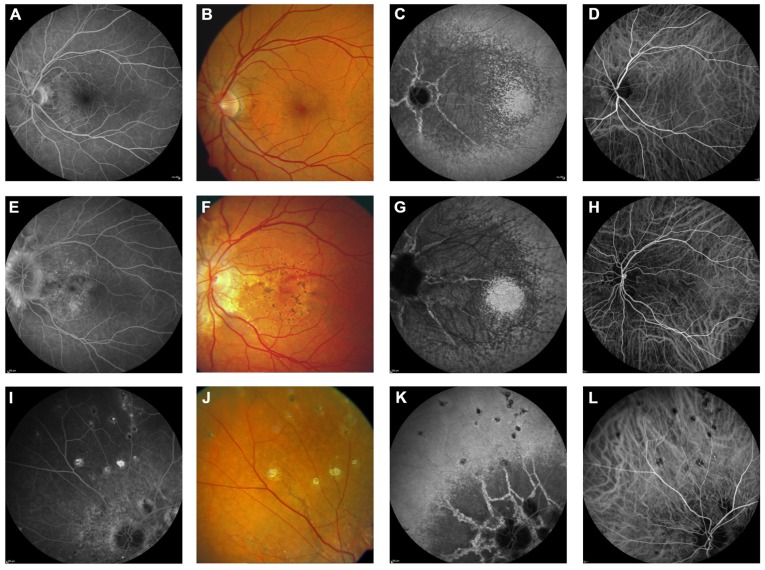
**Comparison of clinical features on late phase fluorescein angiography, early and late phase ICG angiography, and funduscopy** Late phase fluorescein angiography shows variable staining of angioid streaks **(A,E,I)** which corresponds well with findings on funduscopy **(B,F,J)**. A characteristic finding on late phase ICG angiography is a centrally reduced fluorescence with a spotted transition zone to normal peripheral fluorescence **(C,G,K)**. Angioid streaks are well visible within the dark non-fluorescent area. Note that there is no correlate on color images **(B,F,J)** or early ICG angiography frames **(D,H,L)**. Comet tail lesions **(J)** usually are hyperfluorescent on late phase fluorescein angiograms **(I)** and hypofluorescent on ICG late phase angiogram **(K)**.

Comet lesions with or without comet tail (**Figures 1G, 3D**, and **4J**) are observed as solitary, subretinal, nodular, white bodies with a tapering white tail extending posteriorly of the comet body pointing toward the optic disk. The body may have some pigmentation at its margin. Sometimes a spray of comets and comet tails can be observed, creating an aspect of “comet rain.” Comets and comet tails are found in the (mid)periphery of the fundus and are the only PXE-related finding that may occur peripheral to peau d’orange (Gass, 2003; Charbel Issa et al., 2010a). They have been suggested to be the only pathognomonic characteristic of PXE (Gass, 2003) and may also occur in heterozygous carriers of *ABCC6* mutations (De Zaeytijd et al., 2010). Especially in young patients, in whom angioid streaks are often not yet present, they might thus be of significant diagnostic value.

### CHOROIDAL NEOVASCULARIZATION

Choroidal neovascularization of the macular region is a frequent complication in patients with PXE and commonly leads to pronounced vision loss (**Figures 1D,I** and **3F**). Often, CNV in PXE are classic membranes (Nakagawa et al., 2013), i.e., they are located between the RPE and the photoreceptor layer. Classic CNV usually occurs in association with angioid streaks and may develop from occult CNV which is situated underneath the RPE (Nakagawa et al., 2013). The predisposition for the posterior pole is consequent upon the higher frequency of angioid streaks in that area. CNV leads to subretinal hemorrhage and exudation, and eventually formation of a fibrovascular scar (**Figures 1J,K**). Occasionally, eccentric CNV may remain unnoticed due to its lower impact on visual function. Before the development of a scar, CNV is the only ocular PXE complication that is currently eligible for treatment. Intravitreal inhibitors of vascular endothelial growth factor (VEGF) are currently most effective in the attempt to prevent or limit fibrovascular scar formation with consequent visual loss (Gliem et al., 2013). Older treatment options such as photodynamic therapy with verteporfin or argon laser photocoagulation have been largely abandoned. Overall, classic CNV appears to have a worse prognosis with regards to visual function compared to occult CNV (Nakagawa et al., 2013).

Recently, polypoidal choroidal vasculopathy (PCV) was also described to occur in patients with angioid streaks (Baillif-Gostoli et al., 2010; Nakagawa et al., 2013). PCV may occur as an initial vascular change or secondary to CNVs. In contrast to classic CNV, those polyps appear not to be associated with angioid streaks.

### PATTERN DYSTROPHY-LIKE CHANGES

Pattern dystrophy-like changes (**Figures 4F** and **7**) are frequently observed in patients with PXE varying between 10% (Finger et al., 2009a) and almost 70% of cases (Agarwal et al., 2005). Based on a classification suggested by Agarwal and Gass, findings can be categorized due to their similarity to pattern dystrophies, into vitelliform, butterfly, and reticular dystrophy, or fundus flavimaculatus or pulverulentus (von Winning and Oosterhuis, 1974; Shiraki et al., 2001; Agarwal et al., 2005; Sawa et al., 2006; Finger et al., 2009a). It has been postulated that the presence of a pattern dystrophy is a prognostic sign for CNV development although further longitudinal data are needed for confirmation (Finger et al., 2009a).

### CHORIORETINAL ATROPHY

Chorioretinal atrophy may develop secondary to CNVs, usually surrounding a fibrovascular scar. Atrophy can also occur in the absence of CNV (**Figures 7A**–**D**) – a process which, however, has not been well characterized yet. It may initially be observed as patches of atrophy mostly between or along the major vascular arcades, frequently originating within areas of pattern dystrophy. During the subseqent course of the disease, such lesions may grow and – if initially multifocal – become confluent.

### OPTIC NERVE HEAD DRUSEN

Optic nerve head (ONH) drusen (**Figure 1H**) seem to be more common in PXE patients than in the general population. The reported prevalence ranges from 6–8% (Meislik et al., 1979; Finger et al., 2009a) to just over 20% (Pierro et al., 1994) compared to ~0.3% in the general population. To date, it remains unclear why PXE patients are at an increased risk to develop ONH drusen though it may be assumed that a common process of abnormal mineralization might be involved, e.g., through direct calcification or increased rigidity of the lamina cribrosa. Similar to ONH drusen not associated with PXE, ocular ultrasound or fundus autofluorescence (AF) may be required to detect them and may be useful for documentation and follow up (Finger et al., 2009a; De Zaeytijd et al., 2010).

## ADDED VALUE OF RETINAL IMAGING

### OPTICAL COHERENCE TOMOGRAPHY

Spectral domain OCT, which allows for quasi histologic assessment of the posterior ocular fundus *in vivo*, has been used to study PXE-related fundus features (**Figure 2**). On OCT, the calcification of BM may appear as increased reflectivity, and the transition zone from calcified to un-calcified areas correlates well with peau d’orange (Charbel Issa et al., 2009; **Figures 2A**–**E**). Angioid streaks have consistently been found to be associated with breaks in BM (**Figures 2F**–**H**, arrows). There may be differences with regards to the width of the gap, presence or absence of fibrovascular tissue extending through the breaks (**Figures 2I,J**), and alterations or preservations of the overlying RPE layer (Charbel Issa et al., 2009). SD-OCT imaging provided the first direct evidence that breaks in BM are indeed the underlying pathology of angioid streaks (Charbel Issa et al., 2009). Disruption and undulation (inward and outward deformation) of BM on OCT images are much more frequent in eyes of (older) PXE patients than in eyes of AMD patients (Ellabban et al., 2012a), facilitating somewhat the differentiation between those two causes for CNV and chorioretinal atrophy.

Comet tail lesions, the peripheral chorioretinal atrophic spots, may be difficult to record using OCT due to their preferably peripheral localization. The few available scans show hyporeflective spaces involving the outer neurosensory retina with a slightly hyperreflective inner lining and focal debris-like deposits just above the RPE level (Charbel Issa et al., 2009).

Pattern dystrophy-like fundus changes are associated with material deposited below the neurosensory retina, and this may be located either within the RPE layer or just below or above the RPE (Charbel Issa et al., 2009; Zweifel et al., 2011).

Spectral domain OCT has also revealed presence of subretinal fluid in the absence of CNV (**Figures 2N,Q**) in a subset of patients (Zweifel et al., 2011), which may remain undetected by funduscopy. Such fluid accumulation may appear similar to that observed in chronic serous chorioretinopathy. It is probably due to either abnormalities of the RPE pump function, increased hydrophobicity of BM, or a combination thereof. It does not respond to intravitreally applied VEGF inhibitors (Zweifel et al., 2011 and unpublished own observations) or systemic acetazolamide (unpublished own observation). Longstanding cases might develop a vitelliform lesion characterized by deposition of yellowish hyperautofluorescent material at the bottom of the lesion (**Figures 2O**–**Q**).

In daily practice, SD-OCT is a very efficient means to identify neovascular leakage (**Figure 2K**) and monitor treatment efficacy of intravitreal VEGF inhibition. This is of particular importance because it may be difficult in patients with PXE to distinguish between low-grade leakage, which would mean CNV activity, and staining on fluorescein angiography. However, at late stages, atrophic lesions may occur with or without intraretinal cysts (**Figures 2L,M**), which sometimes makes it difficult to distinguish between atrophic retinal cystic alterations and true leakage on OCT, too.

So far there is only little known about pathologic alterations of the choroid in PXE. Histopathologic works reported atrophic changes and disruptions of the choroid in areas of angioid streaks as well as calcification of choroidal vessels (McWilliam, 1951; Dreyer and Green, 1978). Recently, one study measured choroidal thickness in eyes affected by PXE using enhanced depth imaging-OCT and found a reduced choroidal thickness in the subset of eyes that presented with CNV (Ellabban et al., 2012b).

### FLUORESCEIN ANGIOGRAPHY

Fluorescein angiography highlights several features of PXE (**Figure 3**) and, despite the importance of novel techniques such as SD-OCT, still remains the gold standard for detecting and documenting leakage from a CNV in patients with angioid streaks.

Several authors have presented and discussed the fluorescein angiographic findings of angioid streaks (Smith et al., 1964; Patnaik and Malik, 1971; Federman et al., 1975). In the absence of other signs of CNV, however, fluorescein angiography does not usually add clinically relevant information and therefore may be refrained from in asymptomatic patients.

### INDOCYANINE GREEN ANGIOGRAPHY

Indocyanine green angiography uses NIR light for excitation of the chromophore, which is superior to fluorescein angiography in detecting abnormalities under the RPE. ICG differs from fluorescein amongst others in terms of its much more extensive blood-protein binding, and its limited vascular leakage. ICG angiography is capable to outline angioid streaks much better than fluorescein angiography in the majority of cases (Lafaut et al., 1998). Angioid streaks are usually not visible in the early phase, but can be delineated with high sensitivity in the angiographic late phase (**Figure 4**). In the latter, angioid streaks usually are hyperfluorescent within the area of reduced late phase ICG-fluorescence (see below). Outside this area, they may also appear hypofluorescent or invisible in some cases.

**FIGURE 5:**
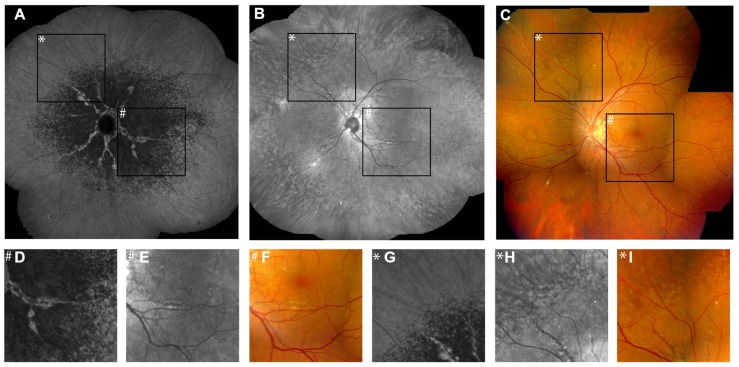
**Comparison of findings on late phase ICG angiography, NIR reflectance imaging and funduscopy**. The transition zone from reduced to normal fluorescence on ICG angiography **(A)** is located more centrally relative to peau d’orange **(B,C)**. Note that there is no correlate of this phenomenon on IR reflection imaging or funduscopy **(D–F)**. Non-invasive near-infrared (NIR) reflectance imaging **(B)** often shows peau d’orange and angioid streaks with greater detail and contrast compared to fundus photography **(C)**. Angioid streaks do not cross peau d’orange **(G–I)**. **(D–I)** Represent a ×3.3 magnification of characteristic details of corresponding areas within **(A–C)**. **(D–F)** Correspond to area #, **(G–I)** correspond to area *.

A highly characteristic PXE-related fundus feature on ICG angiography is a reduced late phase fluorescence centered at the posterior pole (Charbel Issa et al., 2010a), while more eccentric areas exhibit a normal late phase fluorescence. In between these two areas, there is a spotted transition zone, which is most prominent on the temporal side of the fundus in most cases (**Figures 4** and **5**). There are no related obvious alterations on fundus photography, early phase ICG angiography, or fundus AF. Notably, the spotted pattern of peau d’orange is located more eccentric. It was hypothesized that the reduced late phase fluorescence may be due to BM calcification or a dysfunctional RPE (Charbel Issa et al., 2010a).

Indocyanine green is less well suited to detect leakage from CNV compared to fluorescein angiography. Due to its invasive nature it is therefore not recommended to use routinely for monitoring patients but may be used to confirm suspected PCV or occult CNV and to further investigate ocular pathophysiology in PXE.

### FUNDUS AUTOFLUORESCENCE

Fundus AF imaging with most commonly used blue or green excitation light allows evaluation of the integrity and health of the RPE *in vivo*. Many fundus alterations commonly found in PXE may present with characteristic fundus AF abnormalities. Angioid streaks can show areas of increased as well as areas of decreased fundus AF. The latter usually suggests more severe damage of the RPE with cell loss. Wing-like focal spots of increased AF alongside angioid streaks, consisting of pigmentations visible on fundus photography, constitute the parastreak phenomenon (Finger et al., 2009a; De Zaeytijd et al., 2010). Patterns of AF in the macular area in PXE patients are often similar to those observed in patients with pattern dystrophies (**Figure 7**). Comets – and to a lesser extent their tails – typically show a hyperautofluorescent signal. Not all comets can be detected individually on AF imaging (Finger et al., 2009a; De Zaeytijd et al., 2010). Whether this hyperautofluorescence is caused by lipofuscin, or the presence of calcification, or a combination thereof is as yet unclear.

**FIGURE 6:**
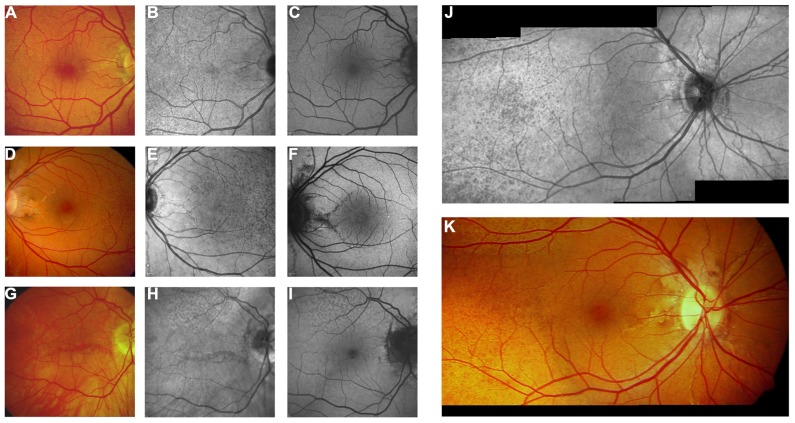
**Near-infrared reflectance imaging and 488 nm fundus autofluorescence in pseudoxanthoma elasticum**. Angioid streaks and peau d’orange are best and most reliably visible on NIR reflectance imaging **(B,E,J)** correlating well with findings on funduscopy **(A,D,K)**. Peau d’orange is usually not discernible on 488 nm fundus autofluorescence images **(C,F)**. Angioid streaks may present with a reduced autofluorescence **(C,F)** but may as well remain undetected on autofluorescence imaging **(H,I)**. Note the reticular drusen on NIR reflectance and 488 nm autofluorescence which are sometimes associated with pseudoxanthoma elasticum **(H,I)**.

Peau d’orange is usually not highlighted on fundus AF images even when visibility was marked on color photography, irrespective of the severity of changes (**Figures 6A**-**I**; Finger et al., 2009a; De Zaeytijd et al., 2010). This seems logical when accepting that the appearance of peau d’orange originates from alterations in Bruch membrane, which is located underneath the RPE (with melanin as strong absorber of short wavelength visible light) and therefore is less effectively imaged using blue light. 

**FIGURE 7:**
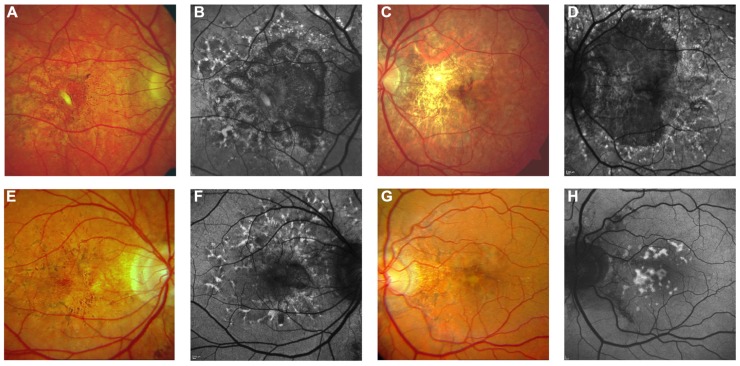
**Pattern dystrophy-like changes and atrophy in pseudoxanthoma elasticum on 488 nm fundus autofluorescence imaging**. Atrophic lesions and pattern dystrophy-like changes are typical features of advanced pseudoxanthoma elasticum. Compared to funduscopic images **(A,C,E,G)** these lesions are best visible on 488 nm fundus autofluorescence images **(B,D,F,H)**. Pattern dystrophy-like lesions encompass different patterns of increased autofluorescence. Depending on the stage of pattern dystrophy changes, atrophy of the retinal pigment epithelium with reduced autofluorescence may be present **(B,D,F)**.

Fundus AF illustrates RPE atrophy as areas of hypo-autofluorescence, which are often more extensive than the areas of atrophy seen on funduscopy (**Figures 7A**–**D**). Thus, fundus AF is useful as a non-invasive tool to monitor progression of RPE changes, including chorioretinal atrophy.

### CONFOCAL REFLECTANCE IMAGING

Confocal NIR reflectance imaging is highly sensitive in detecting peau d’orange and angioid streaks (Charbel Issa et al., 2009; De Zaeytijd et al., 2010). The low absorption rate of 790 nm light by melanin within the RPE, in combination with the high contrast of a confocal imaging system, leads to the superior illustration of such structural alterations underneath the RPE cell layer.

With NIR reflectance imaging, angioid streaks appear as uniform, well-demarcated dark fissures against a lighter background, even when they remain unnoticed on color imaging (**Figure 6**).

Near-infrared reflectance imaging always revealed a diffuse, speckled pattern of peau d’orange, extending beyond the area considered affected on white light digital fundus images. In the absence of extensive macular atrophy or scarring, the peau d’orange area may cover the entire posterior pole and the midperiphery up to the equator (**Figures 5B,C**; Charbel Issa et al., 2009; De Zaeytijd et al., 2010).

Near-infrared reflectance imaging is superior to visualize comets, which went undetected on color images. Comets appear as small white hyperintensities, suggesting high reflectivity for light of NIR wavelengths. 

Recently, confocal NIR reflectance imaging has been shown to be highly sensitive for detecting reticular drusen in patients with age-related macular degeneration. Calcification of Bruch membrane due to PXE also seems to predispose to the development of reticular drusen (**Figures 6H,I**). However, those own preliminary observations have not yet been investigated systematically.

## FUNCTIONAL ALTERATIONS

### VISUAL ACUITY AND VISUAL FIELD

There are very little data available on the natural history of visual acuity loss in PXE, and the same is true for visual field testing. Visual acuity frequently drops to 20/200 or less around the fourth to fifth decade of life and it is not uncommon that only hand movements may be seen (Gliem et al., 2013). Dark adaptation and scotopic perimetry over peau d’orange has not shown significant functional alterations (Holz et al., 1994).

### ELECTROPHYSIOLOGY

Although electrophysiological techniques provide important additional information regarding the underlying causes of visual failure, it has not yet been extensively used to investigate the PXE retinopathy.

Early electrophysiological studies in 15 PXE patients (Francois and De Rouck, 1981) found mild reduced responses on electroretinography (ERG) and electro-oculography (EOG) testing in about 50% of eyes, mostly in those with advanced pathology. Audo et al. (2007) have extended the spectrum of ERG findings in PXE patients suggesting that general retinal dysfunction may occur, which may explain difficulties with night vision, which are frequently reported by PXE patients.

## Histology

In the eye, the first histologically detectable alteration appears to be an abnormal calcification and thickening of the elastic and subsequently of the collagenous layers of BM. Fragmentation and clustering of calcified elastic and collagenous fibres shows similarities to histopathological findings in skin specimen. These changes within BM secondarily may lead to alterations of the adjacent choriocapillaris as well as the overlying RPE and the neurosensory retina. 

Particularly at the posterior pole, BM is thicker than normal and reveals basophilic staining with hematoxylin which is related to calcium deposition. Several authors noted a patchy transition zone towards the rather normal appearing peripheral BM anterior to the equator (Böck, 1938; Hagedoorn, 1939; Klien, 1947; Verhoeff, 1948; Jensen, 1977). This transition zone may be the histopathological correlate for peau d’orange, although no direct evidence has been provided for this interpretation. In late disease stages, the severely altered BM may undergo atrophy (Hagedoorn, 1939).

The histopathological correlate for angioid streaks– breaks in the calcified BM – has been documented in donor eyes of patients with PXE (Böck, 1938; Hagedoorn, 1939; Verhoeff, 1948; Gass, 1967; Jensen, 1977; Dreyer and Green, 1978; Mansour, 1998), Paget’s disease (Gass and Clarkson, 1973), sickle cell disease (Jampol et al., 1987), and of patients without defined underlying disease (Klien, 1947; McWilliam, 1951; Domke and Tost, 1964; Gass, 1967; Dreyer and Green, 1978). Small breaks may remain without morphological changes in the overlying layers of the RPE and retina or of the underlying choriocapillaris. With increasing width of the angioid streaks, RPE cells may become irregular and loose melanin granules. Larger defects in Bruch membrane are often associated with ingrowth of fibrous tissue, RPE cell atrophy and thinning of the choriocapillaris. 

Breaks in BM of PXE patients are a predilection site for the ingrowth of fibrovascular tissue from the choroid. Fibrovascular proliferation may grow underneath the RPE, leaving the detached RPE layer relatively or partially (Böck, 1938; Gass, 1967) intact. Histopathological observations of RPE detachments in patients with angioid streaks have also been described with exudation-like fluid or amorphous material within the sub-RPE space (Böck, 1938; Hagedoorn, 1939; Klien, 1947; Jampol et al., 1987). Frequently, there is very active CNV-proliferation through breaks in BM with subretinal exudation, haemorrhage and subsequent fibrosis, leading to degeneration of the RPE and retina. 

Progressive and diffuse loss of the choriocapillaris paralleling degenerative changes in BM has been noted (Gass, 1967). However, apart from the focal changes along angioid streaks, little attention has been paid to such histopathological changes of the choroid and choriocapillaris. Also, little is known about the histopathological correlate of several clinically described fundus features in patients with PXE. This includes the pattern dystrophy-like changes, subretinal fluid in the absence of CNV, peripheral comet lesion, or reticular drusen. Thus, considering PXE as a well-defined model disease, better knowledge of the PXE-associated ocular pathology may shed light on the pathophysiology of other diseases, such as inherited pattern dystrophy of the retina or age-related macular degeneration. 

## DIFFERENTIAL DIAGNOSIS

The term *PXE-like syndrome* has been used to describe vascular, dermal, and ocular alterations characteristic of PXE that occur secondary to other diseases or due to genetic mutations different from those in *ABCC6*. These include hemoglobinopathies, such as beta-thalassemia or sickle cell disease (Aessopos et al., 2002), Paget’s disease of the bone (Gass and Clarkson, 1973), congenital dyserythropoietic anemia, *ENPP1* mutations causing generalized arterial calcification of infancy (GACI) syndrome (Kalal et al., 2012), and mutations in *GGCX* (Li et al., 2009a). The ocular phenotype described in such patients includes peau d’orange and angioid streaks.

Angioid streaks, the most obvious feature of PXE-related fundus abnormalities, are almost always observed in patients with PXE. The second strongest association appears to be with hemoglobinopathies, including beta-thalassemia and sickle cell disease. Paget’s disease of the bone has been reported to be associated with angioid streaks in 1.4–14% (Scholz, 1941) and peau d’orange in 0–22% of cases (Clarkson and Altman, 1982). It has been reported that angioid streaks may also occur in the absence of any of the PXE-like systemic or ocular alterations (Clarkson and Altman, 1982), including two members of a family with Ehlers–Danlos syndrome (Green et al., 1966). However, up to date phenotyping in such patients would be needed to support such associations.

## SUMMARY AND FUTURE DIRECTIONS

Pseudoxanthoma elasticum may be regarded as a model disease in which calcification of BM leads to a number of secondary effects at the ocular fundus. Of those, the development of CNV is the most vision threatening complication. However, novel therapies using intravitreally applied VEGF inhibitors appear successful in preserving vision over several years (Myung et al., 2010; Finger et al., 2011a; Gliem et al., 2013). Thus, other ocular disease manifestations such as atrophy and/or incompetence of the RPE may be additional future challenges for ophthalmologists caring for PXE patients. In this respect, a better understanding of the pathophysiology leading to several of the classic funduscopic findings is required. For instance, little is currently known on precursors of RPE atrophy and its rate of progression, or the origin of reduced ERG responses in some patients. Finally, it remains to be studied if the ocular phenotype may provide biomarkers that would allow assessing effects of future therapies aiming at systemically reducing soft tissue calcification.

## Conflict of Interest Statement

The authors declare that the research was conducted in the absence of any commercial or financial relationships that could be construed as a potential conflict of interest.
